# In Silico Hydrolysis of Lupin (*Lupinus angustifolius* L.) Conglutins with Plant Proteases Releases Antihypertensive and Antidiabetic Peptides That Are Bioavailable, Non-Toxic, and Gastrointestinal Digestion Stable

**DOI:** 10.3390/ijms252312866

**Published:** 2024-11-29

**Authors:** Jesús Gilberto Arámburo-Gálvez, Raúl Tinoco-Narez-Gil, José Antonio Mora-Melgem, Cesar Antonio Sánchez-Cárdenas, Martina Hilda Gracia-Valenzuela, Lilian Karem Flores-Mendoza, Oscar Gerardo Figueroa-Salcido, Noé Ontiveros

**Affiliations:** 1 Nutrition Sciences Postgraduate Program, Faculty of Nutrition and Gastronomy Sciences, Autonomous University of Sinaloa, Culiacan 80019, Sinaloa, Mexico; gilberto.aramburo@uas.edu.mx (J.G.A.-G.); raultng@gmail.com (R.T.-N.-G.); josemora.uacng@uas.edu.mx (J.A.M.-M.); 2 Integral Postgraduate Program in Biotechnology, Faculty of Chemical and Biological Sciences, Autonomous University of Sinaloa, Ciudad Universitaria, Culiacan 80010, Sinaloa, Mexico; cesar.sanchez@uas.edu.mx; 3 Laboratory for the Research and Detection of Biological Agents and Contaminants (CONAHCYT National Laboratory, LANIBIOC), Yaqui Valley Technological Institute, National Technological Institute of Mexico, Bácum 85276, Sonora, Mexico; martina.gv@vyaqui.tecnm.mx; 4 Clinical and Research Laboratory (LACIUS, C.N., CONAHCYT National Laboratory, LANIBIOC), Deparment of Chemical, Biological, and Agricultural Sciences (DC-QB), Faculty of Biological and Health Sciences, University of Sonora, Navojoa 85880, Sonora, Mexico

**Keywords:** lupin bioactive peptides, ACE-I, DPP-IV, hypertension, diabetes, in silico, bioinformatic, plant proteases

## Abstract

Lupin (*Lupinus angustifolius* L.) proteins are potential sources of bioactive peptides (LBPs) that can inhibit dipeptidyl peptidase IV (DPP-IV) and angiotensin I-converting enzyme (ACE-I) activity. However, the capacity of different enzymes to release LBPs, the pharmacokinetic and bioactivities of the peptides released, and their binding affinities with the active sites of DPP-IV and ECA-I are topics scarcely addressed. Therefore, we used in silico hydrolysis (BIOPEP-UWM platform) with various enzymes to predict the release of LBPs. Among the bioactive peptides identified in lupin proteins (n = 4813), 2062 and 1558 had DPP-IV and ACE-I inhibitory activity, respectively. Ficin, bromelain, and papain released the highest proportion of ACE-I (n = 433, 411, and 379, respectively) and DPP-IV (n = 556, 544, and 596, respectively) inhibitory peptides. LBPs with favorable pharmacokinetics and gastrointestinal stability tightly interacted with the active sites of ACE-I (–5.6 to –8.9 kcal/mol) and DPP-IV (–5.4 to –7.6 kcal/mol). Papain generated the most bioavailable LBPs (n = 459) with ACE-I (n = 223) and DPP-IV (n = 412) inhibitory activity. These peptides were non-toxic and gastrointestinal digestion stable. Notably, papain-based hydrolysis released some LBPs (n = 270) that inhibited both ACE-I and DPP-IV. Plant protease-based hydrolysis is a promising approach for producing lupin hydrolysates with ACE-I and DPP-IV inhibitory activities.

## 1. Introduction

Both type 2 Diabetes Mellitus (DM2) and hypertension commonly coexist and affect a large proportion of the adult population (up to 10.5% and 31.5%, respectively) [1,2]. Around 70% of adult diabetic patients could also have hypertension [3], increasing their risk of developing chronic heart disease and other complications [4]. In these comorbidity cases, angiotensin I-converting enzyme (ACE-I) inhibitors and angiotensin receptor blockers are the drugs of choice for treating hypertension [5]. Likewise, dipeptidyl peptidase-IV (DPP-IV) inhibitors are effective antidiabetic drugs with potential antihypertensive properties [5]. On one hand, ACE-I inhibition avoids the production of the potent vasoconstrictor angiotensin II as well as the cleaving of the vasodilator bradykinin [6], and, on the other hand, DPP-IV inhibition avoids cleaving incretin hormones such as glucose-dependent insulinotropic polypeptide and glucagon-like peptide 1, which help to control plasma glucose levels [7]. Thus, the combination of ACE-I and DPP-IV inhibitors should be considered the primary treatment for diabetic patients with hypertension. However, both ACE-I and DPP-IV inhibitor drugs can cause adverse effects in some patients [8,9], making desirable the use of side effect-free enzyme inhibitors.

DPP-IV and ACE-I inhibitory peptides have been proposed as safe therapeutic options for treating DM2 and hypertension, respectively [10,11,12]. Lupinus species are leguminous plants with high protein content [13] and are sources of ACE-I [14] and DPP-IV [15,16,17] inhibitory peptides. In fact, some peptides released after beta-conglutin hydrolysis with pepsin can inhibit up to 35% of DPP-IV activity [17]. Other lupin peptides can be released with alcalase or flavorzyme. These peptides have shown binding affinities from –8.9 to –10.7 kcal/mol and could inhibit ACE-I [18]. Beyond these findings, some issues related to lupin peptides are worth addressing in depth. Thus, the present study aimed to predict the ACE-I and DPP-IV inhibitory peptides released after conglutin hydrolysis with seven different enzymes (pepsin, trypsin, chymotrypsin, alcalase, papain, bromelain, and ficin) and gastrointestinal digestion to determine their binding affinities with the active sites of DPP-IV and ACE-I and their pharmacokinetic parameters using a comprehensive in silico approach [12,19]. The study is focused on conglutin proteins because they represent 95% of the lupin protein [20].

## 2. Results and Discussion

### 2.1. Biological Activity of Lupin Proteins

The BIOPEP tool “profiles of potential biological activity” was used to search for bioactive peptides encrypted in lupin proteins (α1-3, β1-7, δ1-4, and γ1-2 conglutin). The conglutin family accounts for around 95% of the total protein content of lupin seeds, and it is present in all species and varieties of lupin [21]. A total of 4813 lupin conglutin peptides showed biological activity (Table 1). Among these peptides, 2062 (42.8%) and 1558 (32.3%) showed the potential to inhibit DPP-IV or ACE-I, respectively. Interestingly, the set of peptides generated showed up to 23 distinct bioactivities (Table 1). Others have reported that lupin peptides have osteoprotective, anti-inflammatory, antioxidant, anti-amnesic, anticancer, antibacterial, antidiabetic, and antihypertensive properties [22,23,24]. Thus, our findings support that lupine proteins are sources of peptides with different bioactivities, including a large number of antihypertensive and antidiabetic peptides (DPP-IV and ACE-I inhibitory peptides) [25].

### 2.2. ACE-I and DPP-IV Inhibitory Peptides Released After Simulated Hydrolysis of Lupin Seed Proteins

Among the enzymes utilized to produce protein hydrolysates or peptides with bioactive properties, alcalase (EC 3.4.21.62), bromelain (EC 3.4.22.32), papain (EC 3.4.22.2), and ficin (EC 3.4.22.3) are the most reported [26,27]. These enzymes have distinct cleavage sites and can generate distinct peptide profiles with different bioactive properties [28]. To save time and resources, simulated hydrolysis can serve as a starting point to select an enzyme for searching peptides with specific bioactivities [27,29]. Certainly, bioactive peptides can be generated or broken down during human digestion, making it essential to explore this aspect in silico. In the present study, lupin proteins were hydrolyzed with 8 different enzymes to identify the enzymes with the potential to generate ACE-I or DPP-IV inhibitory peptides. Subsequently, the resulting peptides were subjected to in silico human gastrointestinal digestion (pepsin, trypsin, and chymotrypsin).

**Figure 1:**
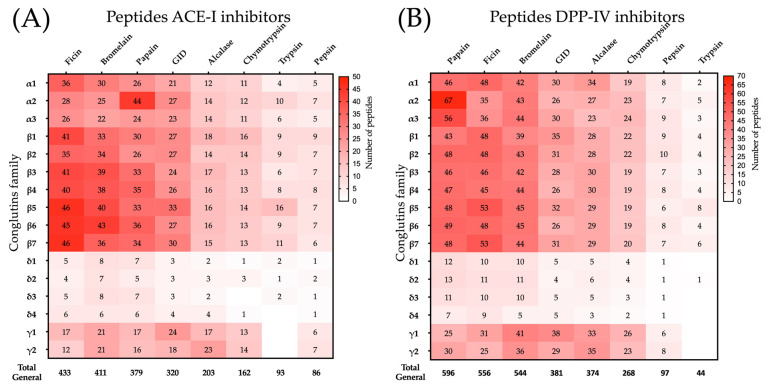
Frequency of conglutin-derived bioactive peptides released after simulated hydrolysis. (**A**) ACE-I inhibitory lupin peptides. (**B**) DPP-IV inhibitory lupin peptides.

Ficin (n = 433), bromelain (n = 411), and papain (n = 379) generated the highest numbers of ACE-I inhibitory peptides after the hydrolysis of the 16 conglutins (Figure 1A). Conversely, chymotrypsin (n = 162), trypsin (n = 93), and pepsin (n = 86) released the lowest numbers of ACE-I inhibitory peptides (Figure 1A). Gastrointestinal digestion (pepsin, trypsin, and chymotrypsin) also generated ACE-I inhibitory peptides (n = 320) (Figure 1A). Similarly, the highest numbers of DPP-IV inhibitory peptides were generated after hydrolysis with papain (n = 596), ficin (n = 556), and bromelain (n = 544), and the lowest ones after hydrolysis with chymotrypsin (n = 268), pepsin (n = 97), and trypsin (n = 44) (Figure 1B). Gastrointestinal digestion also generated DPP-IV inhibitory peptides (n = 381) (Figure 1B).

Overall, the hydrolysis of the 16 conglutins generated unique ACE-I (n = 110) and DPP-IV (n = 151) inhibitory peptides. Similar results were reported using plant proteases and sources other than lupin, such as chickpeas, collagen, and meat proteins [12,19,30,31]. In fact, hydrolysates of various collagen sources obtained with bromelain, papain, or ficin have higher frequencies of DPP-IV and ACE-I inhibitory peptides than hydrolysates obtained with pepsin, trypsin, and chymotrypsin [31]. Additionally, the sequences of some DPP-IV or ACE-I inhibitory synthetic peptides were obtained after in silico digestion of food proteins with plant proteases [32,33,34]. Therefore, plant proteases have the potential to produce antihypertensive and hypoglycemic lupin hydrolysates.

Certainly, the use of synthesized or isolated food-derived peptides may not be cost-effective for treating chronic degenerative diseases, such as hypertension or diabetes [35]. In this context, protein hydrolysates with multiple bioactive peptides have gained attention for developing nutraceuticals or ingredients for functional foods. Among plant proteases, papain, bromelain, and ficin have been used to produce DPP-IV or ACE-I inhibitory food protein hydrolysates [36,37,38,39,40,41]. For instance, chickpea (*Cicer arietinum* L.) protein hydrolysates obtained under optimized hydrolysis conditions with papain or ficin can inhibit DPP-IV in vitro (up to 84.66% and 72.05%, respectively) [41]. Similarly, whey protein hydrolysates obtained using plant proteases (papain, ficin, or bromelain) can inhibit ACE-I in vitro (up to IC50 91.9 μg/mL) [36]. Overall, it is highlighted that plant proteases can be used for producing antidiabetic or antihypertensive food protein hydrolysates, but the DPP-IV and ACE-I inhibitory peptides generated must be stable in gastrointestinal digestion, be absorbed, and remain non-toxic to have the opportunity to be an effective and side effect-free protein hydrolysate.

### 2.3. ADMET Characteristics and Gastrointestinal Digestion Stability of ACE-I and DPP-IV Inhibitory Peptides

The pharmacokinetic properties of the ACE-I and DPP-IV inhibitory peptides identified were evaluated using the ADMET Lab2.0 platform. Table 2 shows the frequency of the LBPs with optimal ADMET prediction values. (For more details, see Supplementary Tables S1 and S2). The ADMET properties were evaluated in 201 unique peptides (151 inhibit DPP-IV (69.90%), 110 inhibit ACE-I (50.92%), and 60 inhibit both enzymes (27.77%)). Almost all ACE-I (94.54%) and all DPP-IV (100%) inhibitory peptides fulfill the Lipinski rule, showing the potential to exert an in vivo effect. Therapeutic compounds designed to be orally administered should be adequately absorbed at the intestinal level and reach systemic circulation. In the present study, almost all peptides identified are potentially absorbed at the intestinal level (ACE-I = 75.45%, DPP-IV = 81.45%) and can reach systemic circulation (ACE-I = 86.36%, DPP-IV = 91.39%). Similarly, almost all the ACE-I and DPP-IV inhibitory peptides identified were short-chain peptides (dipeptides = 83.79%; tripeptides = 15.27%; tetrapeptide = 0.093%), which have higher absorption and bioavailability than long-chain peptides [42]. Additionally, the results show that all the ACE-I and DPP-IV inhibitory peptides identified can be adequately distributed in the systemic circulation, showing that most of them have high half-life times (>3 h) (ACE-I = 86.36%, DPP-IV = 73.51%). Regarding toxicity, most peptides evaluated were non-toxic (ACE-I = 90.91%, DPP-IV = 92.05%).

The stability of the ACE-I and DPP-IV inhibitory peptides during in silico gastrointestinal digestion is shown in Table 2. Most peptides (n = 179; 89.05%) were stable in gastrointestinal digestion. Among these, 97 inhibit DPP-IV, and 82 inhibit ACE-I. The gastrointestinal stability of bioactive peptides depends on several factors, such as peptide length, hydrophobicity, and the presence of specific amino acids (e.g., absence of lysine, presence of proline and aliphatic amino acids) [43]. In this sense, gastrointestinal digestion-stable ACE-I and DPP-IV inhibitory peptides were mostly short-chain peptides (dipeptides = 81.75% and tripeptides = 17.51%). Furthermore, 90.51% of the peptides lack leucine, 63.50% have at least one aliphatic amino acid (e.g., valine, proline, glycine, isoleucine), and 19.70% have proline in their sequence. This explains, to some extent, the in silico gastrointestinal stability of the ACE-I and DPP-IV inhibitory peptides identified in the present study.

### 2.4. Molecular Interactions of Lupin Peptides with the Active Sites of ACE-I and DPP-IV

Molecular docking analysis was performed on all ACE-I (n = 76) and DPP-IV (n = 63) inhibitory peptides with reported PubChem or SatPDB structures. The binding energy of lupin peptides with the active site of ACE-I ranged from –5.4 kcal/mol to –9.2 kcal/mol (Supplementary Table S1). Of them, 51.31% (n = 39) were stable during in silico gastrointestinal digestion and showed optimal ADMET properties. Their binding energies ranged from –5.6 kcal/mol to –8.9 kcal/mol with distances of 1.93–4.83 Å. Among the DPP-IV inhibitory peptides (n = 63), 67.21% (n = 41) were stable in gastrointestinal digestion and had optimal ADMET properties, with binding energies ranging from –5.4 kcal/mol to –7.6 kcal/mol (distances ranged from 1.86–5.53 Å). These binding energies are similar to those reported for other ACE-I (from −5.7 to −9.2 kcal/mol) and DPP-IV (from −5.20 to −8.03 kcal/mol) inhibitory peptides derived from food sources other than lupin (chickpea, saurida elongata, α-lactalbumin, amaranth, and casein) [12,19,44,45,46,47]. Furthermore, the lupin peptides have binding energies similar to the energies reported for synthetic ACE-I (Lisinopril: –8.6 Kcal/mol) [19] and DPP-IV inhibitors (Saxagliptin: –8.4 Kcal/mol and Vildagliptin: –8.8 Kcal/mol) [48,49]. These results highlight that lupin peptides have the potential to tightly bind to the active site of ACE-I and DPP-IV and could act as potential therapeutic inhibitors.

**Figure 2:**
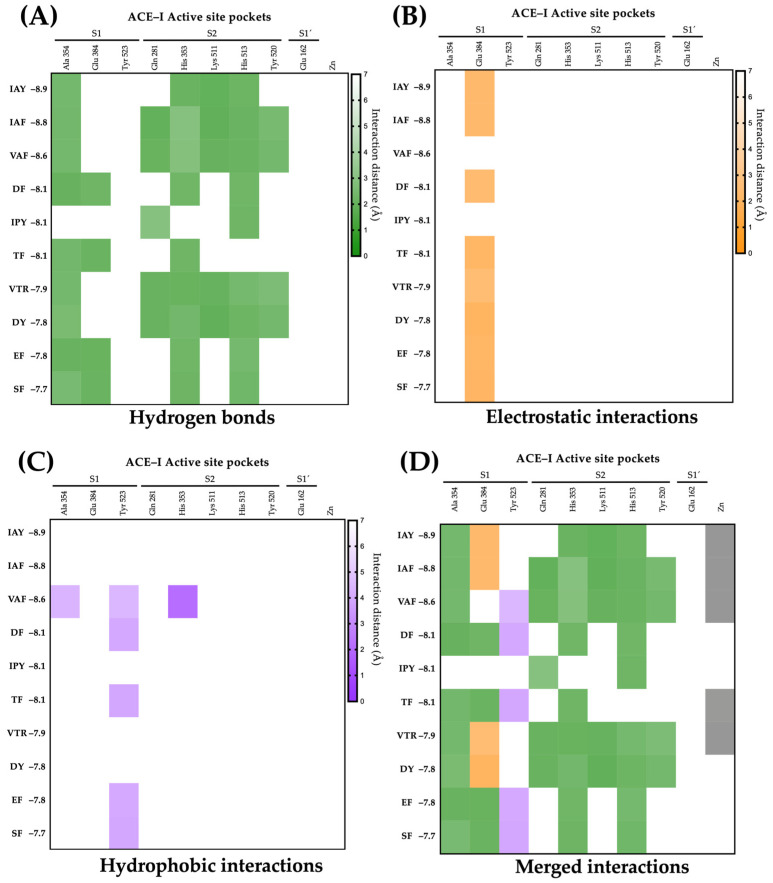
Molecular interactions of conglutin-derived peptides with the active site of ACE-I. (**A**) Hydrogen bonds interactions. (**B**) Electrostatic interactions. (**C**) Hydrophobic interactions. (**D**) Merged interactions.

The active site of ACE-I has three distinct pockets and one cofactor: S1 (Ala354, Glu384, and Tyr523), S2 (Gln281, His353, Lys511, His513, and Tyr520), S1′ (Gln162), and zinc as a cofactor [50]. ACE-I inhibitory peptides could interact with residues of the active site of this enzyme, competitively inhibiting ACE-I [51]. Figure 2 shows the molecular interactions of the ten lupin peptides with the lowest binding energies with the active site residues of ACE-I. ACE-I inhibitory lupin peptides mainly interact with the residues Ala354 (90%) and Glu384 (80%) of pocket S1 via conventional hydrogen bonds and electrostatic interactions at short distances (2.03–3.48 Å) (Figure 2A,B). Five out of 10 ACE-I inhibitory peptides formed hydrophobic interactions with Tyr523 of pocket S1 (distances ranging from 3.95–4.45 Å) (Figure 2C). In contrast, almost all interactions of ACE-I inhibitory peptides with S2 pocket residues were via hydrogen bonds (97.22%) (Figure 2A). Our results support the notion that hydrogen bonds are the main non-covalent interactions mediating the high affinity of ACE-I inhibitory peptides with the active site of ACE-I, but electrostatic and hydrophobic interactions aid in the stabilization of the peptide-ACE-I complex [52]. Interestingly, 5 out of 10 ACE-I inhibitory lupin peptides showed coordination with the zinc ion (Figure 2D). Zinc stabilizes the ACE-I active site, facilitating the coordination of catalytic residues and enabling peptide bonds [53]. Therefore, peptide coordination with zinc ions may induce a higher ACE-I inhibitory activity [54]. Our results show that lupin peptides could interact with residues of the active site of ACE-I, inducing a competitive inhibition.

**Figure 3:**
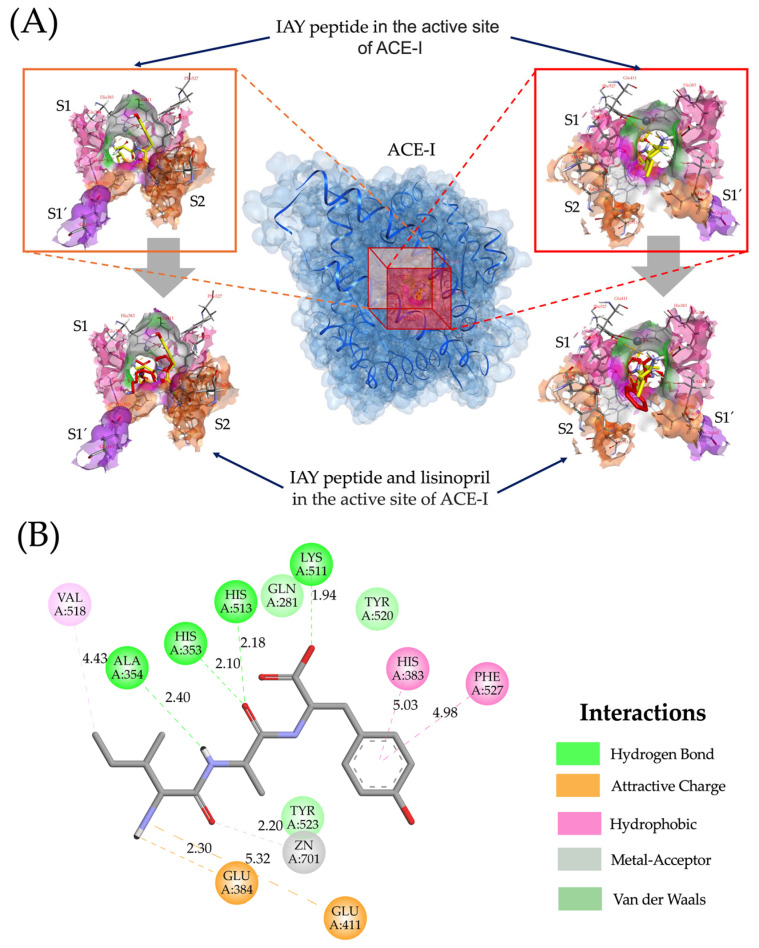
Molecular interactions of the IAY peptide with the active site of ACE-I. (**A**) Three-dimensional representation of the molecular docking of the IAY peptide and its overlapping with lisinopril in the active site of ACE-I. (**B**) Two-dimensional view of the interactions between the IAY peptide and the active site of ACE-I.

Figure 3 shows the molecular interactions of Ile-Ala-Tyr tripeptide in the 3D structure of ACE-I and the overlapping with lisinopril in the ACE-I active site. The tripeptide Ile-Ala-Tyr had the lowest binding energy (−8.9 kcal/mol) and a docking pose comparable to lisinopril (Figure 3A), interacting with residues Ala354 and Glu384 in the S1 pocket via hydrogen bonds and electrostatic interactions (distances < 2.5 Å), as well as with residues His353, Lys511, and His513 in the S2 pocket via hydrogen bonds (Figure 3B). The high affinity of Ile-Ala-Tyr peptide for the active site of ACE-I can be attributed to the presence of Tyr in the C-terminal position. The presence of Tyr and other aromatic residues, such as Trp, Phe, Arg, and His, is associated with a higher ACE-I inhibitory potential among tripeptides, which exhibit the greatest affinity for binding in the ACE-I active site [55]. Additionally, the Ile-Ala-Tyr peptide interacts with zinc via metal coordination, contributing to its high binding affinity [54]. Furthermore, outside the active site, residues His383 and Phe527 stabilize the binding of the tripeptide via hydrophobic interactions, contributing to the negative binding energy (Figure 3B). The molecular interactions of Ile-Ala-Tyr with the active site of ACE-I are similar to those reported for ACE-I synthetic inhibitors, such as captopril and lisinopril [56], highlighting the potential of Ile-Ala-Tyr as a potent ACE-I inhibitor that is stable during in silico gastrointestinal digestion, potentially bioavailable, and non-toxic.

**Figure 4:**
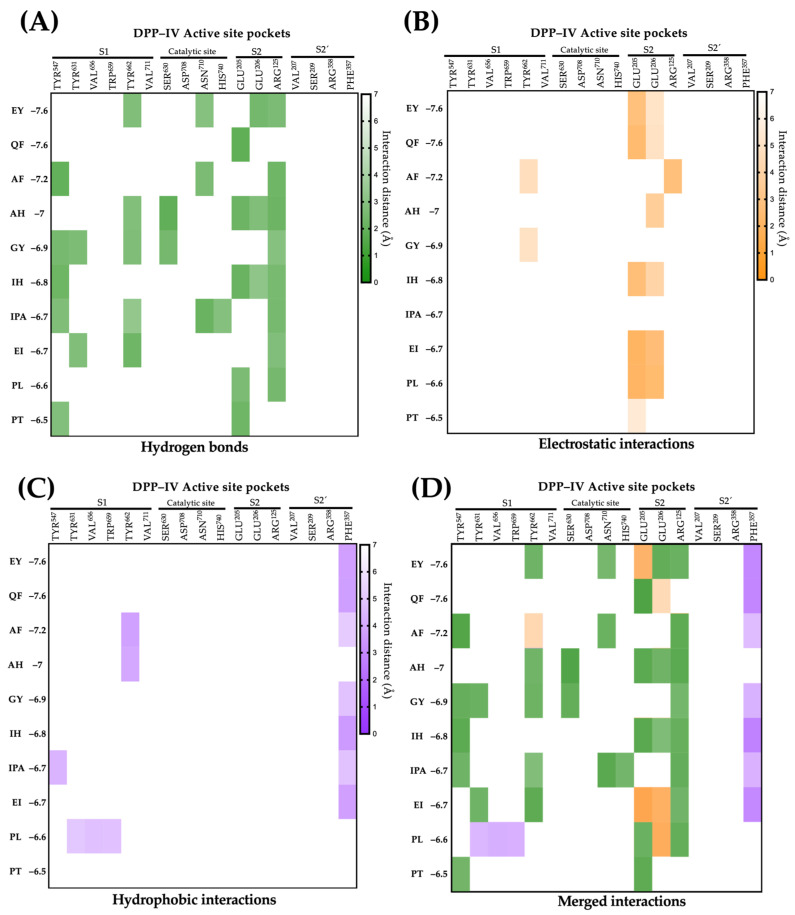
Molecular interactions of conglutin-derived peptides with the DPP-IV active site. (**A**) Hydrogen bonds interactions. (**B**) Electrostatic interactions. (**C**) Hydrophobic interactions. (**D**) Merged interactions.

The DPP-IV active site is composed of four pockets: S1 (Tyr547, Tyr631, Val656, Trp659, Tyr662, and Val711), the catalytic site (Ser630, Asp708, and Asn710), S2 (Glu205, Glu206, and Arg125), and S2′ (Val207, Ser209, Arg358, and Phe357) [57]. The types of and distances of molecular interactions for the ten lupin peptides with the highest affinity for the active site of DPP-IV are shown in Figure 4. Lupin peptides mainly interact with residues of pocket S1 via hydrogen bonds (Tyr437, Tyr 631, and Tyr 662; distances ranging from 1.96 to 2.96 Å) and hydrophobic interactions (Tyr547, Tyr 631, Val656, Trp659, and Tyr662). Most peptides exhibit electrostatic interactions (80%) and hydrogen bonds (70%) with residues of pocket S2. The S1 pocket is characterized by being narrow and favoring interactions with small hydrophobic compounds, while the S2 pocket tends to interact with amino acids via electrostatic interactions [58]. The catalytic site of DPP-IV plays a pivotal role in the inhibition activity of DPP-IV synthetic drugs [59]. The present study shows that lupin peptides interact with residues of the catalytic site of DPP-IV via hydrogen bonds at short distances (1.90–2.95 Å), suggesting their high DPP-IV inhibitory potential. In general, hydrogen bonds were the most frequently non-covalent interactions between lupin peptides and the active site of DPP-IV (55.71%), followed by electrostatic (22.85%) and hydrophobic interactions (21.42%). Others also reported that hydrogen bonds are the main type of non-covalent interactions that mediate the binding of DPP-IV inhibitory lupin peptides with the enzyme’s active site [60]. Overall, the molecular interactions of DPP-IV inhibitory lupin peptides with the enzyme’s active site are similar to those reported for DPP-IV inhibitory synthetic drugs [61].

**Figure 5:**
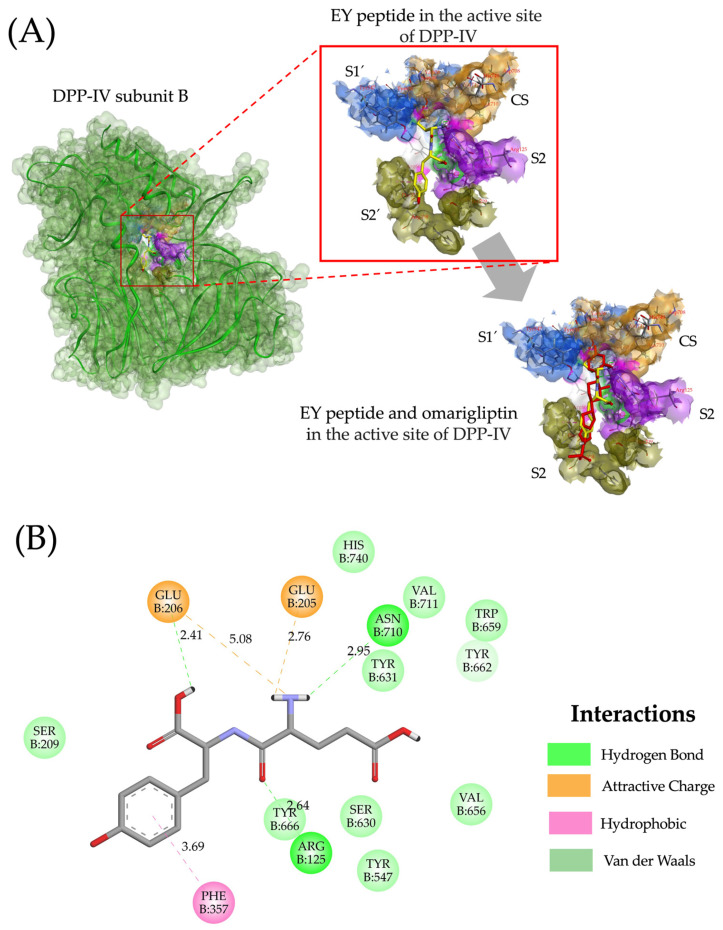
Molecular interactions of the EY peptide with the active site of DPP-IV. (**A**) Three-dimensional representation of the molecular docking of the EY peptide and omarigliptin in the active site of DPP-IV. (**B**) Two-dimensional view of the interactions between the EY peptide and the DPP-IV active site.

Figure 5 shows the molecular interactions of Glu-Tyr dipeptide in the 3D structure of DPP-IV and its overlapping with omarigliptin in the DPP-IV active site. The dipeptide Glu-Tyr had the lowest binding energy (−7.6 kcal/mol) among the DPP-IV inhibitory peptides and had a docking pose similar to omarigliptin (Figure 5A). The Glu-Tyr peptide forms various interactions with the DPP-IV active site: it interacts with residues Tyr662 in the S1 pocket, Asn710 in the catalytic triad, and with Glu206 and Arg125 in the S2 pocket via hydrogen bonds (Figure 5A). Additionally, it establishes electrostatic interactions with residues Glu205 and Glu206 in the S2 pocket and forms hydrophobic interactions with the residue Phe357 in the S2′ pocket (Figure 5B). These interactions contribute to explaining the low binding energy of the ligand–receptor (peptide–protein) complex [62]. Additionally, Glu-Tyr peptide shares similarities with omarigliptin, binding with residues Tyr662, Glu205, Glu206, and Arg125 [63] and interacting with the same residues (Arg125, Glu205, Glu206, Phe357, Tyr662, and Asn710) that the tripeptide IPI interacts with in the active site of DPP-IV [64], a tripeptide that has the lowest reported IC_50_ value (3.5 μM) [62].

### 2.5. Plant Proteases Generate Lupine Protein Hydrolysates Containing Bioavailable, Non-Toxic, Digestion-Resistant, and Multi-Bioactive Peptides

Once the peptides’ pharmacokinetic properties, gastrointestinal digestion stability, and molecular interactions with the ACE-I and DPP-IV active sites were determined, the peptides were grouped according to their protein source and the enzyme that released them. This approach allows the evaluation of all peptides organized as hydrolysates. Supplementary Tables S3–S10 show the number of lupin peptides released after simulated hydrolysis. These peptides can inhibit DPP-IV, ACE-I, or both enzymes. The bioavailability, toxicity, and gastrointestinal digestion stability of the peptides from each hydrolysate are also shown in Tables S3–S10. Optimal peptides were those predicted to be bioavailable, non-toxic, and digestion resistant. Ficin (n = 717), papain (n = 705), and bromelain (n = 703) generated the hydrolysates with the highest number of ACE-I or DPP-IV inhibitory peptides. The number of optimal peptides in the other hydrolysates ranged from 115 to 547.

**Figure 6:**
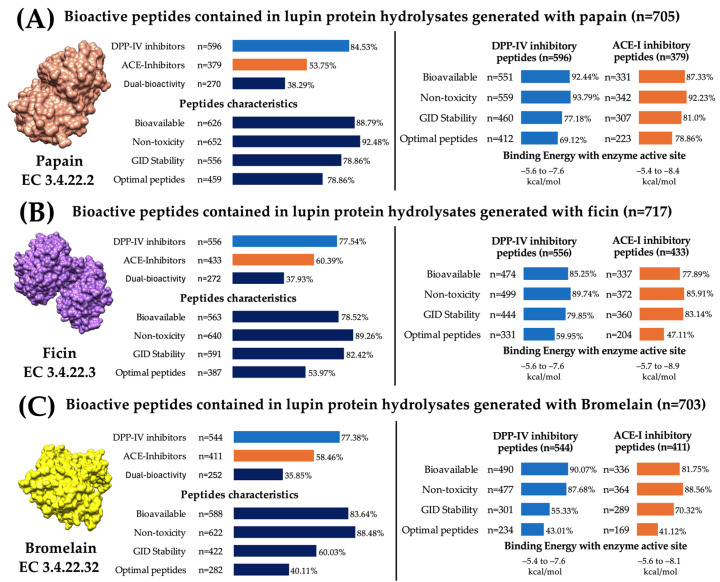
Characteristics of plant protease-based lupin protein hydrolysates. (**A**) Characteristics of papain hydrolysates. (**B**) Characteristics of ficin hydrolysates. (**C**) Characteristics of bromelain hydrolysates. Bioavailable (F20% < 0.3); non-toxicity (ROAT < 0.3). GID: gastrointestinal digestion; optimal peptides: bioavailable, non-toxic, and stable in GID.

Despite having similar cleavage sites, plant proteases generated lupin protein hydrolysates containing peptides with different pharmacokinetic and bioactive characteristics (Figure 6). Papain generates the hydrolysate with the highest number of optimal peptides (n = 459). Among these, 89.76% (n = 412) were DPP-IV inhibitory peptides with binding energies ranging from −6.5 to −7.6 kcal/mol, 48.58% (n = 223) were ACE-I inhibitory peptides with binding energies ranging from −5.4 to −8.4. kcal/mol, and 38.34% (n = 176) had the potential to inhibit both enzymes (Figure 6A). Ficin generates hydrolysates containing 387 optimal bioactive peptides. Among these, 85.5% (n = 331) were DPP-IV inhibitory peptides with binding energies from −5.6 to 7.6, 52.71% (n = 204) were ACE-I inhibitory peptides with binding energies from −5.7 to −8.9, and 38.24% (n = 148) had the potential to inhibit both enzymes (Figure 6B). Regarding bromelain, this plant protease generates lupin protein hydrolysates with optimal peptides (n = 282). Among these, 82.97% (n = 234) were DPP-IV inhibitory peptides with binding energies from –5.4 to –7.6, 59.92% (n = 169) were ACE-I inhibitory peptides with binding energies from –5.6 to –8.1, and 42.9% (n = 121) had the potential to inhibit both enzymes (Figure 6C). These results show that lupine protein hydrolysates with antihypertensive and hypoglycemic properties can be obtained using plant proteases. These enzymes can work over wide pH and temperature ranges, but their use in producing hydrolysates containing ACE-I and DPP-IV inhibitory peptides remains largely unexplored [65]. Particularly, papain, compared to ficin and bromelain, has specific cleavage sites at both the C-terminus and N-terminus of peptide bonds [32], generating a wide variety of bioactive peptides that can not only inhibit DPP-IV and ACE-I but could also interact with other therapeutic targets.

## 3. Materials and Methods

### 3.1. Protein Sequences and Virtual Screening for Bioactive Peptides

Protein sequences of lupin storage proteins were obtained from the UniProtKB database (Conglutin alpha 1; Uni-Prot ID: F5B8V6, Conglutin alpha 2; UniProt ID: F5B8V7, Conglutin alpha 3; UniProt ID: F5B8V8, Conglutin beta 1; UniProt ID: F5B8V9, Conglutin beta 2; UniProt ID: F5B8W0, Conglutin beta 3; UniProt ID: F5B8W1, Conglutin beta 4; UniProt ID: F5B8W2, Conglutin beta 5; UniProt ID: F5B8W3, Conglutin beta 6; UniProt ID: F5B8W4, Conglutin beta 7; UniProt ID: F5B8W5, Gamma Conglutin 1; UniProt ID: Q42369, Gamma Conglutin 2; UniProt ID: F5B8W7, Conglutin Delta 1; UniProt ID: F5B8W8, Conglutin Delta 2; UniProt ID: Q99235, Conglutin Delta 3; UniProt ID: F5B8X0, Conglutin Delta 4; UniProt ID: F5B8X1).

Virtual screening for bioactive peptides was performed according to previous studies with minor modifications [12,19]. Briefly, the “Bioactive Peptides” tab was selected to perform the analyses in the BIOPEP-UWM platform (University of Warmia and Mazury in Olsztyn, Olsztyn, Poland) [66]. Afterwards, the tool “profiles of potential biological activity” available in the “Analysis” tab was utilized to determine the bioactive peptide sequences within lupin proteins. In silico enzymatic hydrolysis was performed utilizing the BIOPEP-UWM platform using the following enzymes: pepsin (EC 3.4.23.1), trypsin (EC 3.4.21.4), chymotrypsin (EC 3.4.21.1), alcalase (EC 213 3.4.21.62), papain (3.4.22.2), bromelain (3.4.22.32), and ficin (EC 3.4.22.3). Simulated gastrointestinal digestion was carried out using pepsin (EC 3.4.23.1), trypsin (EC 3.4.21.4), and chymotrypsin (EC 3.4.21.1), simultaneously. The potential biological activity of peptides released was screened using the BIOPEP-UWM database, which enables searching for 68 different biological activities of proteins/peptides.

### 3.2. ADMET Properties and Gastrointestinal Digestion Stability

The pharmacokinetic properties of ACE-I and DPP-IV inhibitory peptides were evaluated using the ADMETLab 2.0 platform (Zhejiang University, Hangzhou, China), as previously described [12,19]. The following pharmacokinetic properties were evaluated: (1) Lipinski’s rule, (2) human intestinal absorption, (3) volume of distribution, (4) half-life, and (5) acute oral toxicity in rats. The criteria available in the ADMETLab 2.0 platform were used to interpret the pharmacokinetic predictions. Optimal ADMET was considered when a peptide was predicted to be bioavailable (F20% < 0.3) and non-toxic (ROAT < 0.3). Gastrointestinal digestion stability of ACE-I and DPP-IV inhibitory peptides with optimal ADMET values was assessed using the BIOPEP-UWM platform (pepsin (EC 3.4.23.1), trypsin (EC 3.4.21.4), and chymotrypsin (EC 3.4.21.1)).

### 3.3. Molecular Docking of ACE-I and DPP-IV Inhibitory Peptides

The crystallographic structure of human ACE-I (complex with lisinopril; PDB ID: 1O86) and DPP-IV (complex with omarigliptin; PDB ID: 4PNZ) were obtained from the Protein Data Bank. Molecular docking analyses were carried out when the three-dimensional structures of ACE-I and DPP-IV inhibitory peptides were available in the PubChem Database. All structures were prepared for molecular docking (peptides: adding polar hydrogens and charges; ACE-I and DPP-IV crystallographic structures: (1) adding polar hydrogens and charges and (2) removal of water molecules and crystallographic ligands). Docking was performed using the AutoDock Vina 1.1.2. (The Scripps Research Institute, CA, USA). The coordinates selected for docking analysis covers the active site of ACE-I (x: 40.6559, y: 37.3827, z: 43.3401, radius: 20 Å) and DPP-IV (x: −6.733, y: 62.839, z: 35.416, radius: 20 Å) [12,19]. The following parameters were used for molecular docking analyses: number of binding modes per ligand = 10, exhaustiveness = 8, and maximum energy difference between models = 2 kcal/mol. The best docking pose showing the lowest binding energy for each predicted peptide was selected. Dockings between peptides and the active sites of ACE-I and DPP-IV were visualized using the Discovery Studio software v21.1.0 (Dassault Systèmes, Vélizy-Villacoublay, France). Dockings with unfavorable interactions were excluded.

## 4. Conclusions

Lupin conglutins are sources of ACE-I and DPP-IV inhibitory peptides. These peptides are mainly released after the hydrolysis of conglutins with plant proteases. Papain, ficin, and bromelain showed the best yields to produce ACE-I and DPP-IV inhibitory peptides. The peptides with the strongest binding energy in the active sites of DPP-IV and ECA-I were Glu-Tyr and Ile-Ala-Tyr, respectively. These peptides were bioavailable, non-toxic, and gastrointestinal digestion stable. It should be highlighted that the ACE-I and DPP-IV inhibitory potential of lupin peptides remain to be evaluated in vitro and in vivo to corroborate their bioactivity. However, our findings serve as the groundwork for future studies aimed at evaluating in vitro and in vivo the antihypertensive and hypoglycemic potential of plant protease-based lupin protein hydrolysates.

## Figures and Tables

**Table 1:** Number of conglutin-derived peptides with specific bioactivities.

Bioactivity	Lupin Conglutin Families																Total General
	α1	α2	α3	β1	β2	β3	β4	β5	β6	β7	δ1	δ2	δ3	δ4	γ1	γ2	
DPP-IV inhibitor	148	147	158	155	151	150	151	149	149	149	55	109	55	40	149	147	2062
ACE-I inhibitor	132	116	112	122	119	116	117	117	117	118	37	72	37	33	92	101	1558
DPP III inhibitor	21	23	19	24	23	21	21	23	22	22	7	11	7	2	21	20	287
Antioxidative	26	20	23	21	17	20	19	19	19	17	5	11	6	3	21	21	268
Stimulating	11	12	9	12	12	11	11	11	11	11	7	14	7	5	10	8	162
Renin inhibitor	12	8	8	11	11	9	9	9	10	10	1	2	1		6	7	114
Alpha-glucosidase inhibitor	3	5	6	5	4	4	4	6	4	5			1	1	1	5	54
Regulating	3	4	2	3	4	4	4	5	4	4	1	2	1		3	3	47
Antithrombotic	5	2	2	4	3	2	2	3	2	3	2	4	2		2	2	40
Anti-inflammatory	2	4	4	3	3	3	3	3	3	3			1		2	2	36
Neuropeptide	2	3	3	3	3	2	3	3	3	3				1	1	3	33
CaMPDE inhibitor	3	2	2	3	3	3	3	3	3	3					1	2	31
Activating ubiquitin-mediated proteolysis	2	2	2	2	2	1	1	2	1	2	1	2	1	1	3	3	28
Anti-amnestic	2	2	1	2	2	2	2	2	2	2	1	2	1		3	2	28
Hypocholesterolemic		2		3	1	3	1	1	1								12
Hypolipidemic	1	1	1	1	1	1	1	1	1	1						1	11
Immunomodulating		1	1	1	1	1	1	1	1	1					1		10
HMG-CoA reductase inhibitor		1	1	1	1	1				1					1	1	8
Hypotensive	1			1							1	2	1	1			7
Anticancer		1		1	1	1				1							5
Bacterial permease ligand		1		1										1		1	4
Immunostimulating			1					1								1	3
Antibacterial	1	1															2
Embryotoxic	1		1														2
Opioid																1	1
Total general	376	358	356	379	362	355	353	359	353	356	118	231	121	88	317	331	4813

**Table 2:** Frequency of conglutin-derived ACE-I and DPP-IV inhibitory peptides with optimal ADMET properties and gastrointestinal digestion stability.

Parameters	General (n = 201)		DPP-IV (n = 151)		ACE-I (n = 110)	
	n	%	n	%	n	%
HIA	157	78.10	123	81.46	83	75.45
F20%	176	87.56	138	91.39	95	86.36
F30%	164	81.59	128	84.77	90	81.82
VD	201	100.00	151	100.00	110	100.00
T ½	156	77.61	111	73.51	95	86.36
ROAT	184	91.56	139	92.05	100	90.91
LIPINSKI	191	95.02	104	68.87	104	94.55
GDS	179	89.05	97	64.23	82	74.54

Optimal ADMET was considered when a peptide was predicted to be bioavailable (F20% < 0.3) and non-toxic (ROAT < 0.3). HIA: human intestinal absorption; F20%: oral bioavailability 20%; F30%: oral bioavailability 30%; VD: volume of distribution; T ½: half-life time; ROAT: rat oral acute toxicity; GDS: gastrointestinal digestion stability.

## Data Availability

The original contributions presented in the study are included in the article/Supplementary Material, further inquiries can be directed to the corresponding author/s. PDB format of peptide-enzyme complex and 2D interactions of molecular docking for the peptides presented in Figure 2 and Figure 4 are available in a freely accessible repository (https://doi.org/10.6084/m9.figshare.27143631, accessed on 1 October 2024).

## Supplementary Materials

The following supporting information can be downloaded at: https://www.mdpi.com/article/10.3390/ijms252312866/s1.
