# Supplementary material for: In Silico Hydrolysis of Lupin (Lupinus angustifolius L.) Conglutins with Plant Proteases Releases Antihypertensive and Antidiabetic Peptides That Are Bioavailable, Non-Toxic, and Gastrointestinal Digestion Stable
Source: Int J Mol Sci. 2024 Nov 29;25(23):12866. doi: 10.3390/ijms252312866 (PMC11641171; doi:10.3390/ijms252312866)
Supplement: Supplementary file 1 [file ijms-25-12866-s001.zip › ijms-3265720-supplementary.pdf]

Supplementary Material Table S1. ADMET properties, gastrointestinal digestion stability and binding energy of ACE-I inhibitory lupin peptides.

Peptide	PubChem ID	HIA	F(20%)	F(30%)	VDss	T 1/2	ROA	Lipinski	Optimal ADMET properties	Gastrointestinal Digestion Stability	Binding Enegy (Kcal/Mol)
AF	ID 6992394	0.022	0.002	0.001	0.249	0.869	0.154	Accepted	YES	YES	-7.5
AG	-	0.007	0.003	0.001	0.284	0.774	0.024	Accepted	YES	YES	-
AGS	CID 135103	0.078	0.017	0.028	0.553	0.818	0.002	Accepted	YES	YES	-6.1
AH	CID 9837455	0.016	0.003	0.002	0.341	0.916	0.052	Accepted	YES	YES	-6.5
AR	ID 446132	0.012	0.004	0.003	0.506	0.395	0.054	Accepted	YES	YES	-6.2
AY	-	0.014	0.002	0.001	0.296	0.887	0.102	Accepted	YES	YES	-
CF	CID 25051327	0.209	0.582	0.022	0.255	0.901	0.454	Accepted	NO	YES	-7.3
DA	CID 5491963	0.021	0.003	0.001	0.261	0.822	0.005	Accepted	YES	YES	-5.6
DF	CID 93078	0.106	0.003	0.004	0.234	0.845	0.045	Accepted	YES	YES	-8.1
DG	ID 151148	0.016	0.004	0.004	0.274	0.782	0.011	Accepted	YES	YES	-5.8
DM	ID satpdb11083	0.016	0.003	0.001	0.291	0.865	0.009	Accepted	YES	YES	-5.7
DR	CID 16122509	0.908	0.751	0.417	0.819	0.542	0.008	Accepted	NO	YES	-6.9
DY	CID 152455	0.042	0.004	0.013	0.303	0.877	0.04	Accepted	YES	YES	-7.8
EA	ID 6992506	0.064	0.003	0.002	0.275	0.842	0.003	Accepted	YES	YES	-5.7
EF	CID 18653118	0.222	0.004	0.01	0.248	0.868	0.032	Accepted	YES	YES	-7.8
EG	ID 6427052	0.039	0.004	0.011	0.279	0.8	0.007	Accepted	YES	YES	-5.9
EI	CID 9813855	0.457	0.004	0.002	0.309	0.874	0.014	Accepted	YES	YES	-6.3
EK	ID 7015703	0.82	0.013	0.387	0.502	0.774	0.013	Accepted	YES	YES	-6.4
EQR	-	0.559	0.997	0.999	0.499	0.766	0.006	Rejected	NO	YES	-

ER	CID 9796450	0.283	0.788	0.977	0.442	0.81	0.015	Accepted	NO	YES	-6.4
EV	ID 6992567	0.055	0.003	0.002	0.286	0.85	0.011	Accepted	YES	YES	-6.1
EW	-	0.045	0.003	0.025	0.244	0.903	0.134	Accepted	YES	YES	-
EY	515717	0.101	0.007	0.031	0.314	0.891	0.029	Accepted	YES	YES	-7.5
FDK	-	0.932	0.065	0.567	0.369	0.873	0.075	Accepted	YES	NO	-
GF	CID 92953	0.161	0.009	0.006	0.284	0.886	0.045	Accepted	YES	YES	-7.2
GH	-	0.044	0.014	0.012	0.377	0.931	0.039	Accepted	YES	YES	-
GI	ID 88079	0.027	0.003	0.002	0.523	0.913	0.026	Accepted	YES	YES	-5.6
GK	-	0.473	0.031	0.025	0.713	0.805	0.029	Accepted	YES	YES	-
GL	ID 1548899	0.012	0.002	0.001	0.446	0.874	0.03	Accepted	YES	YES	-6.1
GPL	-	0.505	0.008	0.006	0.44	0.838	0.02	Accepted	YES	YES	-
GPM	CID 67746222	0.223	0.029	0.008	0.383	0.893	0.007	Accepted	YES	YES	-6.8
GR	-	0.098	0.864	0.826	0.633	0.792	0.034	Accepted	NO	YES	-
GS	-	0.076	0.023	0.042	0.78	0.857	0.005	Accepted	YES	YES	-
GW	-	0.058	0.008	0.022	0.353	0.921	0.17	Accepted	YES	YES	-
GY	ID 92829	0.069	0.199	0.033	0.357	0.895	0.06	Accepted	YES	YES	-7.2
HG	-	0.021	0.004	0.005	0.328	0.916	0.088	Accepted	YES	NO	-
HHL	-	0.026	0.004	0.012	0.347	0.942	0.124	Rejected	YES	NO	-
HK	CID 148224	0.307	0.008	0.048	0.506	0.904	0.107	Accepted	YES	NO	-6.3
HL	CID 189008	0.013	0.002	0.001	0.402	0.919	0.17	Accepted	YES	NO	-7
HY	CID 515713	0.072	0.008	0.008	0.326	0.922	0.2	Accepted	YES	NO	-8.2
IA	CID 7009577	0.023	0.004	0.001	0.35	0.849	0.026	Accepted	YES	YES	-5.7
IAF	CID 656985	0.063	0.004	0.001	0.247	0.873	0.13	Accepted	YES	YES	-8.8
IAK	CID 10042290	0.804	0.011	0.004	0.515	0.823	0.048	Accepted	YES	YES	-7.5
IAY	CID 11610285	0.039	0.004	0.002	0.253	0.885	0.07	Accepted	YES	YES	-8.9

IF	CID 7009595	0.025	0.003	0.001	0.254	0.873	0.621	Accepted	NO	YES	-7.8
IG	ID 6992869	0.016	0.003	0.001	0.31	0.791	0.151	Accepted	YES	YES	-5.7
IL	ID 7019083	0.009	0.002	0.001	0.39	0.851	0.314	Accepted	NO	YES	-6.5
IP	CID 444876	0.3	0.004	0.004	0.373	0.778	0.034	Accepted	YES	YES	-6.3
IPA	CID 10040393	0.599	0.006	0.004	0.336	0.854	0.013	Accepted	YES	YES	-7.4
IPY	CID 14889694	0.263	0.006	0.005	0.239	0.893	0.086	Accepted	YES	YES	-8.1
IQY	-	0.072	0.189	0.521	0.301	0.723	0.054	Accepted	YES	YES	-
IR	CID 7021814	0.028	0.004	0.002	0.532	0.384	0.106	Accepted	YES	YES	-6.7
IVF	-	0.02	0.004	0.001	0.262	0.876	0.394	Accepted	NO	YES	-
IY	CID 7408194	0.022	0.004	0.002	0.284	0.9	0.47	Accepted	NO	YES	-8
KA	ID 7010504	0.568	0.005	0.003	0.594	0.78	0.025	Accepted	YES	NO	-5.8
KF	CID 151410	0.849	0.01	0.067	0.415	0.835	0.235	Accepted	YES	NO	-7.4
KG	ID 7022320	0.246	0.006	0.025	0.619	0.709	0.084	Accepted	YES	NO	-5.7
KL	-	0.135	0.002	0.001	0.607	0.799	0.137	Accepted	YES	NO	-
KR	-	0.714	0.205	0.985	0.667	0.754	0.092	Accepted	YES	NO	-
KY	-	0.86	0.067	0.167	0.446	0.868	0.206	Accepted	YES	NO	-
LR	-	0.008	0.002	0.001	0.549	0.399	0.102	Accepted	YES	NO	-
MG	-	0.01	0.002	0.001	0.313	0.871	0.029	Accepted	YES	NO	-
NF	-	0.078	0.006	0.014	0.23	0.599	0.05	Accepted	YES	NO	-
NG	-	0.008	0.007	0.009	0.283	0.52	0.011	Accepted	YES	NO	-
NK	CID 11219156	0.07	0.05	0.547	0.555	0.504	0.017	Accepted	YES	NO	-6.2
NY	-	0.045	0.025	0.102	0.289	0.691	0.039	Accepted	YES	NO	-
PG	CID 6426709	0.611	0.132	0.498	0.609	0.766	0.047	Accepted	YES	YES	-5.8
PH	CID 9856353	0.047	0.524	0.989	0.5	0.887	0.11	Accepted	NO	YES	-7.2
PL	ID 444109	0.244	0.002	0.003	0.52	0.816	0.158	Accepted	YES	YES	-6.9

PPK	CID 9881087	0.953	0.752	0.98	0.622	0.786	0.055	Accepted	NO	YES	-7.1
PQR	CID 145457442	0.918	0.999	0.999	0.482	0.714	0.078	Rejected	NO	YES	-8.2
PR	CID 151004	0.846	0.979	0.993	0.614	0.788	0.096	Accepted	NO	YES	-7
PSY	CID 145457580	0.74	0.997	0.999	0.417	0.836	0.073	Accepted	NO	YES	-9
PT	CID 53860028	0.076	0.144	0.043	0.596	0.881	0.012	Accepted	YES	YES	-6.3
QDVL	-	0.419	0.348	0.145	0.396	0.75	0.011	Rejected	NO	YES	-
QG	-	0.008	0.01	0.047	0.285	0.52	0.008	Accepted	YES	YES	-
QK	-	0.187	0.1	0.86	0.547	0.5	0.017	Accepted	YES	YES	-
QP	CID 11736661	0.092	0.018	0.06	0.32	0.491	0.005	Accepted	YES	YES	-6.5
RF	CID 150964	0.595	0.411	0.918	0.368	0.824	0.168	Accepted	NO	NO	-8.4
RL	CID 6992563	0.008	0.002	0.001	0.56	0.424	0.106	Accepted	YES	NO	-7.2
RW	CID 7009653	0.416	0.393	0.991	0.39	0.871	0.346	Accepted	NO	NO	-9.1
RY	CID 7021456	0.48	0.98	0.984	0.395	0.83	0.128	Accepted	NO	NO	-8.4
SF	CID 7009597	0.559	0.004	0.01	0.683	0.805	0.029	Accepted	YES	YES	-7.7
SG	CID 7009644	0.093	0.006	0.009	0.913	0.761	0.02	Accepted	YES	YES	-5.4
ST	CID 54027404	0.023	0.02	0.012	0.394	0.903	0.003	Accepted	YES	YES	-5.7
SY	-	0.231	0.004	0.015	0.777	0.849	0.025	Accepted	YES	YES	-
TF	CID 7010580	0.026	0.002	0.002	0.419	0.803	0.06	Accepted	YES	YES	-8.1
TG	-	0.014	0.005	0.003	0.596	0.775	0.029	Accepted	YES	YES	-
TQ	-	0.022	0.005	0.006	0.342	0.815	0.007	Accepted	YES	YES	-
VAF	ID satpdb14951	0.021	0.004	0.001	0.252	0.859	0.089	Accepted	YES	YES	-8.6
VE	ID 7009623	0.03	0.003	0.002	0.279	0.825	0.011	Accepted	YES	YES	-6.3
VF	ID 6993120	0.013	0.002	0.001	0.254	0.872	0.5	Accepted	NO	YES	-7.8
VG	ID 6993110	0.007	0.003	0.001	0.304	0.775	0.083	Accepted	YES	YES	-5.7
VIY	-	0.025	0.006	0.002	0.249	0.893	0.263	Accepted	YES	YES	-

VK	-	0.276	0.004	0.002	0.599	0.732	0.098	Accepted	YES	YES	-
VM	CID 6993039	0.01	0.002	0.001	0.375	0.867	0.042	Accepted	YES	YES	-6
VP	CID 9837272	0.09	0.003	0.003	0.376	0.744	0.031	Accepted	YES	YES	-6.1
VR	-	0.01	0.003	0.001	0.529	0.377	0.099	Accepted	YES	YES	-
VSP	CID 118709853	0.946	0.022	0.086	0.444	0.835	0.009	Accepted	YES	YES	-7.6
VTR	CID 10177411	0.025	0.02	0.012	0.498	0.523	0.014	Rejected	YES	YES	-7.9
VVF	CID 7014911	0.019	0.003	0.001	0.26	0.872	0.316	Accepted	NO	YES	-9.2
VVL	CID 44230570	0.048	0.003	0.002	0.377	0.835	0.109	Accepted	YES	YES	-6.9
VW	-	0.01	0.002	0.002	0.25	0.901	0.746	Accepted	NO	YES	-
VY	ID 7009554	0.011	0.003	0.001	0.274	0.887	0.401	Accepted	NO	YES	-7.8
YG	ID 7021853	0.03	0.006	0.012	0.272	0.867	0.155	Accepted	YES	NO	-7.2
YK	CID 7021830	0.863	0.033	0.123	0.432	0.862	0.131	Accepted	YES	NO	-7.2
YL	CID 87071	0.008	0.002	0.001	0.299	0.909	0.277	Accepted	YES	NO	-7.9
YN	ID 44230566	0.041	0.021	0.109	0.282	0.687	0.031	Accepted	YES	NO	-7.4
YNL	CID 16222279	0.026	0.05	0.007	0.279	0.747	0.057	Accepted	YES	NO	-7.9
YPR	-	0.877	0.976	0.985	0.336	0.843	0.115	Rejected	NO	NO	-

Supplementary Material Table S2. ADMET properties, gastrointestinal digestion stability and binding energy of DPP-IV inhibitory lupin peptides.

Peptide	PubChem ID	HIA	F(20%)	F(30%)	VDss	T12	ROA	Lipinski	Optimal ADMET properties	Gastrointestinal Digestion Stability	Binding Enegy (Kcal/Mol)
AF	ID 6992394	0.022	0.002	0.001	0.249	0.869	0.154	Accepted	YES	YES	-7.2
AG	-	0.007	0.003	0.001	0.284	0.774	0.024	Accepted	YES	YES	-
AH	CID 9837455	0.016	0.003	0.002	0.341	0.916	0.052	Accepted	YES	YES	-7
AL	-	0.009	0.002	0.001	0.349	0.848	0.052	Accepted	YES	YES	-
AT	-	0.016	0.007	0.004	0.452	0.896	0.006	Accepted	YES	YES	-
AY	-	0.014	0.002	0.001	0.296	0.887	0.102	Accepted	YES	YES	-
DN	-	0.014	0.023	0.021	0.35	0.611	0.004	Accepted	YES	YES	-
DP	-	0.744	0.004	0.006	0.258	0.784	0.006	Accepted	YES	YES	-
DR	CID 16122509	0.908	0.751	0.417	0.819	0.542	0.008	Accepted	NO	YES	-6.8
EG	ID 6427052	0.039	0.004	0.011	0.279	0.8	0.007	Accepted	YES	YES	-6
EH	-	0.044	0.005	0.015	0.307	0.923	0.021	Accepted	YES	YES	-
EI	CID 9813855	0.457	0.004	0.002	0.309	0.874	0.014	Accepted	YES	YES	-6.6
EK	ID 7015703	0.82	0.013	0.387	0.502	0.774	0.013	Accepted	YES	YES	-6.2
ES	ID 6995653	0.411	0.012	0.47	0.453	0.884	0.002	Accepted	YES	YES	-6.2
ET	ID 6998031	0.022	0.006	0.008	0.302	0.929	0.002	Accepted	YES	YES	-6.3
EV	-	0.055	0.003	0.002	0.286	0.85	0.011	Accepted	YES	YES	-
EW	-	0.045	0.003	0.025	0.244	0.903	0.134	Accepted	YES	YES	-
EY	515717	0.101	0.007	0.031	0.314	0.891	0.029	Accepted	YES	YES	-7.6

GF	-	0.161	0.009	0.006	0.284	0.886	0.045	Accepted	YES	YES	-
GH	-	0.044	0.014	0.012	0.377	0.931	0.039	Accepted	YES	YES	-
GI	ID 88079	0.027	0.003	0.002	0.523	0.913	0.026	Accepted	YES	YES	-5.4
GL	ID 1548899	0.012	0.002	0.001	0.446	0.874	0.03	Accepted	YES	YES	-5.7
GPM	-	0.223	0.029	0.008	0.383	0.893	0.007	Accepted	YES	YES	-
GW	-	0.058	0.008	0.022	0.353	0.921	0.17	Accepted	YES	YES	-
GY	ID 92829	0.069	0.199	0.033	0.357	0.895	0.06	Accepted	YES	YES	-6.9
HA	ID 101180	0.017	0.003	0.002	0.338	0.914	0.022	Accepted	YES	NO	-6.3
HF	CID 152198	0.114	0.003	0.006	0.323	0.92	0.211	Accepted	YES	NO	-7.7
HL	CID 189008	0.013	0.002	0.001	0.402	0.919	0.17	Accepted	YES	NO	-6.8
HR	-	0.087	0.25	0.936	0.442	0.892	0.115	Accepted	YES	NO	-
HS	-	0.064	0.01	0.037	0.562	0.908	0.011	Accepted	YES	NO	-
HT	-	0.018	0.015	0.007	0.63	0.941	0.012	Accepted	YES	NO	-
HY	CID 515713	0.072	0.008	0.008	0.326	0.922	0.2	Accepted	YES	NO	-8.2
IA	CID 7009577	0.023	0.004	0.001	0.35	0.849	0.026	Accepted	YES	YES	-5.8
IH	CID 7019081	0.017	0.004	0.003	0.354	0.916	0.235	Accepted	YES	YES	-6.8
IL	ID 7019083	0.009	0.002	0.001	0.39	0.851	0.314	Accepted	NO	YES	-6.3
IM	CID 7020106	0.016	0.002	0.001	0.386	0.881	0.041	Accepted	YES	YES	-5.6
IN	CID 7016080	0.009	0.007	0.002	0.333	0.52	0.015	Accepted	YES	YES	-5.9
IP	CID 444876	0.3	0.004	0.004	0.373	0.778	0.034	Accepted	YES	YES	-5.8
IPA	CID 10040393	0.599	0.006	0.004	0.336	0.854	0.013	Accepted	YES	YES	-6.7
IQ	CID 7020102	0.016	0.008	0.003	0.356	0.52	0.01	Accepted	YES	YES	-6.2
IR	CID 7021814	0.028	0.004	0.002	0.532	0.384	0.106	Accepted	YES	YES	-6.1
KA	ID 7010504	0.568	0.005	0.003	0.594	0.78	0.025	Accepted	YES	NO	-5.8
KF	CID 151410	0.849	0.01	0.067	0.415	0.835	0.235	Accepted	YES	NO	-7

KG	ID 7022320	0.246	0.006	0.025	0.619	0.709	0.084	Accepted	YES	NO	-5.9
KH	-	0.299	0.011	0.063	0.511	0.904	0.14	Accepted	YES	NO	-
KR	-	0.714	0.205	0.985	0.667	0.754	0.092	Accepted	YES	NO	-
KS	-	0.832	0.04	0.231	0.792	0.815	0.012	Accepted	YES	NO	-
KT	-	0.236	0.051	0.017	0.614	0.886	0.015	Accepted	YES	NO	-
KV	-	0.392	0.003	0.002	0.621	0.788	0.092	Accepted	YES	NO	-
KY	-	0.86	0.067	0.167	0.446	0.868	0.206	Accepted	YES	NO	-
MA	ID 7009581	0.012	0.002	0.001	0.368	0.886	0.01	Accepted	YES	NO	-5.1
MG	-	0.01	0.002	0.001	0.313	0.871	0.029	Accepted	YES	NO	-
MH	CID 7408323	0.024	0.002	0.002	0.355	0.924	0.062	Accepted	YES	NO	-6
ML	-	0.01	0.001	0.001	0.414	0.887	0.067	Accepted	YES	NO	-
MQ	-	0.014	0.005	0.006	0.364	0.669	0.006	Accepted	YES	NO	-
MR	ID 25216498	0.018	0.005	0.004	0.524	0.514	0.053	Accepted	YES	NO	-6.5
MV	-	0.011	0.002	0.001	0.413	0.896	0.035	Accepted	YES	NO	-
NA	-	0.009	0.006	0.002	0.296	0.572	0.004	Accepted	YES	NO	-
ND	-	0.015	0.026	0.024	0.354	0.608	0.004	Accepted	YES	NO	-
NF	-	0.078	0.006	0.014	0.23	0.599	0.05	Accepted	YES	NO	-
NG	-	0.008	0.007	0.009	0.283	0.52	0.011	Accepted	YES	NO	-
NH	-	0.025	0.009	0.046	0.318	0.831	0.03	Accepted	YES	NO	-
NL	-	0.006	0.004	0.001	0.326	0.556	0.018	Accepted	YES	NO	-
NM	-	0.011	0.004	0.003	0.33	0.662	0.007	Accepted	YES	NO	-
NN	-	0.01	0.227	0.184	0.343	0.407	0.005	Accepted	YES	NO	-
NP	CID 9920984	0.027	0.015	0.019	0.307	0.49	0.006	Accepted	YES	NO	-6.2
NQ	-	0.01	0.212	0.564	0.347	0.404	0.003	Accepted	YES	NO	-
NR	ID 14299174	0.612	0.909	0.89	0.788	0.422	0.009	Accepted	NO	NO	-6.9

NT	-	0.016	0.023	0.007	0.357	0.758	0.003	Accepted	YES	NO	-
NV	-	0.007	0.005	0.002	0.325	0.55	0.013	Accepted	YES	NO	-
NY	-	0.045	0.025	0.102	0.289	0.691	0.039	Accepted	YES	NO	-
PA	ID 6347578	0.705	0.006	0.01	0.485	0.818	0.02	Accepted	YES	YES	-5.4
PF	6351946	0.431	0.594	0.978	0.461	0.823	0.452	Accepted	NO	YES	-6.8
PG	CID 6426709	0.611	0.132	0.498	0.609	0.766	0.047	Accepted	YES	YES	-5.6
PH	CID 9856353	0.047	0.524	0.989	0.5	0.887	0.11	Accepted	NO	YES	-7
PI	-	0.672	0.006	0.014	0.554	0.842	0.17	Accepted	YES	YES	-
PK	CID 9209431	0.84	0.48	0.865	0.723	0.739	0.078	Accepted	NO	YES	-5.8
PL	ID 444109	0.244	0.002	0.003	0.52	0.816	0.158	Accepted	YES	YES	-6.6
PM	CID 7408172	0.219	0.003	0.006	0.475	0.843	0.032	Accepted	YES	YES	-6
PN	CID 7408193	0.205	0.855	0.915	0.467	0.546	0.015	Accepted	NO	YES	-6.7
PS	CID 7408258	0.48	0.89	0.98	0.494	0.809	0.015	Accepted	NO	YES	-6
PT	CID 53860028	0.076	0.144	0.043	0.596	0.881	0.012	Accepted	YES	YES	-6.5
PV	-	0.41	0.004	0.008	0.523	0.812	0.084	Accepted	YES	YES	-
PY	ID 152264	0.197	0.965	0.991	0.595	0.862	0.442	Accepted	NO	YES	-7.1
QA	-	0.011	0.006	0.003	0.323	0.569	0.003	Accepted	YES	YES	-
QD	-	0.019	0.052	0.119	0.348	0.621	0.003	Accepted	YES	YES	-
QE	-	0.044	0.058	0.564	0.367	0.652	0.001	Accepted	YES	YES	-
QF	57288566	0.202	0.01	0.078	0.249	0.61	0.042	Accepted	YES	YES	-7.6
QG	-	0.008	0.01	0.047	0.285	0.52	0.008	Accepted	YES	YES	-
QH	-	0.037	0.015	0.21	0.329	0.835	0.028	Accepted	YES	YES	-
QI	-	0.021	0.006	0.003	0.387	0.594	0.014	Accepted	YES	YES	-
QL	-	0.007	0.004	0.002	0.356	0.556	0.014	Accepted	YES	YES	-
QN	-	0.01	0.291	0.596	0.351	0.402	0.004	Accepted	YES	YES	-

QP	CID 11736661	0.092	0.018	0.06	0.32	0.491	0.005	Accepted	YES	YES	-6.3
QQ	-	0.011	0.353	0.872	0.364	0.395	0.002	Accepted	NO	YES	-
QS	-	0.046	0.109	0.205	0.517	0.711	0.002	Accepted	YES	YES	-
QT	-	0.017	0.033	0.012	0.38	0.761	0.002	Accepted	YES	YES	-
QV	-	0.009	0.004	0.002	0.357	0.547	0.01	Accepted	YES	YES	-
QY	-	0.099	0.078	0.472	0.296	0.695	0.033	Accepted	YES	YES	-
RH	-	0.09	0.465	0.96	0.449	0.892	0.135	Accepted	NO	NO	-
RI	-	0.039	0.004	0.002	0.573	0.426	0.137	Accepted	YES	NO	-
RL	-	0.008	0.002	0.001	0.56	0.424	0.106	Accepted	YES	NO	-
RN	-	0.038	0.899	0.988	0.475	0.619	0.028	Accepted	NO	NO	-
RW	-	0.416	0.393	0.991	0.39	0.871	0.346	Accepted	NO	NO	-
SF	-	0.559	0.004	0.01	0.683	0.805	0.029	Accepted	YES	YES	-
SH	CID 7016094	0.218	0.006	0.017	0.61	0.876	0.019	Accepted	YES	YES	-6.2
SK	CID 16122513	0.741	0.034	0.105	0.558	0.829	0.017	Accepted	YES	YES	-5.7
SL	CID 7015694	0.017	0.003	0.001	0.368	0.871	0.02	Accepted	YES	YES	-5.9
SY	-	0.231	0.004	0.015	0.777	0.849	0.025	Accepted	YES	YES	-
TF	-	0.026	0.002	0.002	0.419	0.803	0.06	Accepted	YES	YES	-
TG	-	0.014	0.005	0.003	0.596	0.775	0.029	Accepted	YES	YES	-
TH	-	0.02	0.003	0.005	0.462	0.885	0.03	Accepted	YES	YES	-
TK	-	0.441	0.006	0.008	0.63	0.715	0.021	Accepted	YES	YES	-
TL	-	0.022	0.005	0.004	0.472	0.8	0.032	Accepted	YES	YES	-
TM	-	0.05	0.004	0.003	0.32	0.875	0.011	Accepted	YES	YES	-
TN	-	0.02	0.005	0.005	0.315	0.83	0.016	Accepted	YES	YES	-
TQ	-	0.022	0.005	0.006	0.342	0.815	0.007	Accepted	YES	YES	-
TR	-	0.025	0.006	0.01	0.569	0.411	0.024	Accepted	YES	YES	-

TS	-	0.048	0.011	0.063	0.737	0.826	0.004	Accepted	YES	YES	-
TY	-	0.021	0.002	0.003	0.492	0.838	0.048	Accepted	YES	YES	-
VA	CID 6992637	0.008	0.004	0.001	0.373	0.798	0.031	Accepted	YES	YES	-5.6
VE	-	0.03	0.003	0.002	0.279	0.825	0.011	Accepted	YES	YES	-
VF	-	0.013	0.002	0.001	0.254	0.872	0.5	Accepted	NO	YES	-
VG	-	0.007	0.003	0.001	0.304	0.775	0.083	Accepted	YES	YES	-
VH	CID 7408625	0.015	0.003	0.002	0.357	0.912	0.185	Accepted	YES	YES	-6.3
VI	ID 7010531	0.011	0.004	0.002	0.428	0.865	0.43	Accepted	NO	YES	-5.7
VK	-	0.276	0.004	0.002	0.599	0.732	0.098	Accepted	YES	YES	-
VL	CID 6993117	0.011	0.002	0.001	0.39	0.836	0.235	Accepted	YES	YES	-6.2
VM	-	0.01	0.002	0.001	0.375	0.867	0.042	Accepted	YES	YES	-
VN	CID 7020201	0.007	0.005	0.002	0.309	0.498	0.017	Accepted	YES	YES	-6.1
VP	-	0.09	0.003	0.003	0.376	0.744	0.031	Accepted	YES	YES	-
VPL	CID 7408179	0.03	0.003	0.003	0.364	0.832	0.038	Accepted	YES	YES	-6.2
VQ	CID 7016045	0.008	0.005	0.002	0.332	0.506	0.011	Accepted	YES	YES	-6.1
VR	-	0.01	0.003	0.001	0.529	0.377	0.099	Accepted	YES	YES	-
VS	CID 6992640	0.05	0.004	0.005	0.674	0.817	0.009	Accepted	YES	YES	-5.7
VT	CID 9815826	0.014	0.009	0.005	0.559	0.893	0.014	Accepted	YES	YES	-6.1
VV	ID 107475	0.012	0.003	0.002	0.395	0.839	0.204	Accepted	YES	YES	-5.6
VW	-	0.01	0.002	0.002	0.25	0.901	0.746	Accepted	NO	YES	-
VY	-	0.011	0.003	0.001	0.274	0.887	0.401	Accepted	NO	YES	-
WF	ID 6426942	0.392	0.006	0.009	0.185	0.915	0.513	Accepted	NO	NO	-8.6
WR	-	0.383	0.165	0.989	0.38	0.868	0.295	Accepted	YES	NO	-
WV	-	0.009	0.002	0.002	0.292	0.918	0.729	Accepted	NO	NO	-
YF	CID 7009600	0.286	0.009	0.011	0.181	0.921	0.417	Accepted	NO	NO	-7.5

YG	-	0.03	0.006	0.012	0.272	0.867	0.155	Accepted	YES	NO	-
YI	CID 7019109	0.024	0.003	0.001	0.312	0.926	0.402	Accepted	NO	NO	-7.1
YK	-	0.863	0.033	0.123	0.432	0.862	0.131	Accepted	YES	NO	-
YL	-	0.008	0.002	0.001	0.299	0.909	0.277	Accepted	YES	NO	-
YN	-	0.041	0.021	0.109	0.282	0.687	0.031	Accepted	YES	NO	-
YR	CID 123804	0.465	0.969	0.981	0.383	0.831	0.108	Accepted	NO	NO	-7.3
YS	-	0.081	0.018	0.131	0.742	0.861	0.017	Accepted	YES	NO	-
YT	-	0.02	0.008	0.004	0.627	0.914	0.022	Accepted	YES	NO	-

Supplementary Material Table S3. Characteristics of bioactive peptides contained in lupin protein hydrolysates generated with papain.

Conglutins family proteins hydrolysates with papain	Total peptides n=705	ACE-I inhibition n=379	DPP-IV inhinition n=596	Dual-activity n=270
		**Peptide characteristics**		
**Bioavailable**	88.79% (n=626)	87.34% (n=331)	92.45% (n=551)	94.81% (n=256)
**Non-toxicity**	92.48% (n=652)	90.24% (n=342)	93.79% (n=559)	92.22% (n=249)
**GID Stability**	78.87% (n=556)	81.0% (n=307)	77.18% (n=460)	78.15% (n=211)
**Optimal peptides**	65.11% (n=459)	58.84% (n=223)	69.13% (n=412)	65.19% (n=176)
**Binding Energy with enzyme active site (kcal/mol)**	–	–5.6 to –7.6	–5.4 to –8.4	–

Bioavailable (F20% <0.3); Non-toxicity (ROAT <0.3). GID: gastrointestinal digestion; Optimal peptides: bioavailable, non-toxic and stable to GID.

Supplementary Material Table S4. Characteristics of bioactive peptides contained in lupin protein hydrolysates generated with ficin.

Conglutins family proteins hydrolysates with ficin	Total peptides n=717	ACE-I inhibition n=433	DPP-IV inhinition n=556	Dual-activity n=272
		**Peptide characteristics**		
**Bioavailable**	78.54% (n=563)	77.83% (n=337)	85.25% (n=474)	91.18% (n=248)
**Non-toxicity**	89.26% (n=640)	85.91% (n=372)	89.75% (n=499)	84.93% (n=231)
**GID Stability**	82.43% (n=591)	83.14% (n=360)	79.86% (n=444)	78.31% (n=213)
**Optimal peptides**	53.97% (n=387)	47.11% (n=204)	59.53% (n=331)	54.41% (n=148)
**Binding Energy with enzyme active site (kcal/mol)**	–	–5.6 to –7.6	–5.7 to –8.9	–

Bioavailable (F20% <0.3); Non-toxicity (ROAT <0.3). GID: gastrointestinal digestion; Optimal peptides: bioavailable, non-toxic and stable to GID.

Supplementary Material Table S5. Characteristics of bioactive peptides contained in lupin protein hydrolysates generated with bromelain.

Conglutins family proteins hydrolysates with bromelain	Total peptides	ACE-I inhibition	DPP-IV inhinition	Dual-activity
	n=703	n=411	n=544	n=252
	Peptide characteristics			
**Bioavailable**	83.64% (n=588)	81.75% (n=336)	90.07% (n=490)	94.44% (n=238)
**Non-toxicity**	88.48% (n=622)	88.56% (n=364)	87.68% (n=477)	86.9% (n=219)
**GID Stability**	60.03% (n=422)	70.32% (n=289)	55.33% (n=301)	66.67% (n=168)
**Optimal peptides**	40.11% (n=282)	41.12% (n=169)	43.01% (n=234)	48.02% (n=121)
**Binding Energy with enzyme active site (kcal/mol)**		–5.4 to –7.6	–5.6 to –8.1	

Bioavailable (F20% <0.3); Non-toxicity (ROAT <0.3). GID: gastrointestinal digestion; Optimal peptides: bioavailable, non-toxic and stable to GID.

Supplementary Material Table S6. Characteristics of bioactive peptides contained in lupin protein hydrolysates generated with alcalase.

Conglutins family proteins hydrolysates with alcalase	Total peptides	ACE-I inhibition	DPP-IV inhinition	Dual-activity
	n=418	n=203	n=374	n=159
	Peptide characteristics			
**Bioavailable**	94.5% (n=395)	98.03% (n=199)	94.65% (n=354)	99.37% (n=158)
**Non-toxicity**	82.3% (n=344)	68.97% (n=140)	82.35% (n=308)	65.41% (n=104)
**GID Stability**	78.71% (n=329)	84.24% (n=171)	77.54% (n=290)	83.02% (n=132)
**Optimal peptides**	57.18% (n=239)	53.69% (n=109)	55.61% (n=208)	49.06% (n=78)
**Binding Energy with enzyme active site (kcal/mol)**		–5.6 to –8.8	–5.4 to –7.6	

Bioavailable (F20% <0.3); Non-toxicity (ROAT <0.3). GID: gastrointestinal digestion; Optimal peptides: bioavailable, non-toxic and stable to GID.

Supplementary Material Table S7. Characteristics of bioactive peptides contained in lupin protein hydrolysates generated with gastrointestinal digestion (GID).

Conglutins family proteins hydrolysates with GID	Total peptides	ACE-I inhibition	DPP-IV inhinition	Dual-activity
	n=547	n=320	n=381	n=154
	**Peptide characteristics**			
**Bioavailable**	68.37% (n=374)	58.75% (n=188)	82.68% (n=315)	83.77% (n=129)
**Non-toxicity**	89.21% (n=488)	86.88% (n=278)	86.61% (n=330)	77.92% (n=120)
**GID Stability**	100% (n=547)	100% (n=320)	100% (n=381)	100% (n=154)
**Optimal peptides**	60.88% (n=333)	45.94% (n=147)	73.75% (n=281)	61.69% (n=95)
**Binding Energy with enzyme active site (kcal/mol)**		–5.7 to –8.8	–5.6 to –7.6	

Bioavailable (F20% <0.3); Non-toxicity (ROAT <0.3). GID: gastrointestinal digestion; Optimal peptides: bioavailable, non-toxic and stable to GID.

Supplementary Material Table S8. Characteristics of bioactive peptides contained in lupin protein hydrolysates generated with pepsin.

Conglutins family proteins hydrolysates with pepsin	Total peptides	ACE-I inhibition	DPP-IV inhinition	Dual-activity
	n=122	n=86	n=97	n=61
		**Peptide characteristics**		
**Bioavailable**	97.54% (n=119)	98.84% (n=85)	97.94% (n=95)	100.0% (n=61)
**Non-toxicity**	83.61% (n=102)	79.07% (n=68)	86.6% (n=84)	81.97% (n=50)
**GID Stability**	76.23% (n=93)	76.74% (n=66)	71.13% (n=69)	68.85% (n=42)
**Optimal peptides**	59.84% (n=73)	55.81% (n=48)	57.73% (n=56)	50.82% (n=31)
**Binding Energy with enzyme active site (kcal/mol)**		–6.1 to –8.8	–7.2	

Bioavailable (F20% <0.3); Non-toxicity (ROAT <0.3). GID: gastrointestinal digestion; Optimal peptides: bioavailable, non-toxic and stable to GID.

Supplementary Material Table S9. Characteristics of bioactive peptides contained in lupin protein hydrolysates generated with chymotrypsin.

Conglutins family proteins hydrolysates with chymotrypsin	Total peptides	ACE-I inhibition	DPP-IV inhinition	Dual-activity
	n=307	n=162	n=268	n=123
		**Peptide characteristics**		
**Bioavailable**	81.76% (n=251)	92.59% (n=150)	80.6% (n=216)	93.5% (n=115)
**Non-toxicity**	82.74% (n=254)	75.93% (n=123)	83.21% (n=223)	74.8% (n=92)
**GID Stability**	79.15% (n=243)	76.54% (n=124)	77.24% (n=207)	71.54% (n=88)
**Optimal peptides**	57.33% (n=176)	47.53% (n=77)	55.97% (n=150)	41.46% (n=51)
**Binding Energy with enzyme active site (kcal/mol)**		–5.7 to –8.8	–7.2	

Bioavailable (F20% <0.3); Non-toxicity (ROAT <0.3). GID: gastrointestinal digestion; Optimal peptides: bioavailable, non-toxic and stable to GID.

Supplementary Material Table S10. Characteristics of bioactive peptides contained in lupin protein hydrolysates generated with trypsin.

Conglutins family proteins hydrolysates with Trypsin	Total peptides	ACE-I inhibition	DPP-IV inhinition	Dual-activity
	n=115	n=93	n=44	n=22
		Peptide characteristics		
**Bioavailable**	44.35% (n=51)	39.78% (n=37)	75.0%% (n=33)	86.36% (n=19)
**Non-toxicity**	100.0% (n=115)	100.0% (n=93)	100.0% (n=44)	100.0% (n=22)
**GID Stability**	73.04% (n=84)	87.1% (n=81)	52.27% (n=23)	90.91% (n=20)
**Optimal peptides**	24.35% (n=28)	26.88% (n=25)	45.45% (n=20)	77.27% (n=17)
**Binding Energy with enzyme active site (kcal/mol)**		–6.4 to –6.7	–7.2	

Bioavailable (F20% <0.3); Non-toxicity (ROAT <0.3). GID: gastrointestinal digestion; Optimal peptides: bioavailable, non-toxic and stable to GID.
